# Antimicrobial Efficiency of Chitosan and Its Methylated Derivative against *Lentilactobacillus parabuchneri* Biofilms

**DOI:** 10.3390/molecules27248647

**Published:** 2022-12-07

**Authors:** Diellza Bajrami, Stephan Fischer, Holger Barth, Syed Imdadul Hossain, Nicola Cioffi, Boris Mizaikoff

**Affiliations:** 1 Institute of Analytical and Bioanalytical Chemistry, Ulm University, Albert Einstein-Allee 11, 89081 Ulm, Germany; 2 Institute of Pharmacology and Toxicology, Ulm University Medical Center, Albert Einstein-Allee 11, 89081 Ulm, Germany; 3 Chemistry Department, University of Bari “Aldo Moro”, Via E. Orabona, 4, 70126 Bari, Italy; 4 CSGI (Center for Colloid and Surface Science) c/o Chemistry Department, Via E. Orabona, 4, 70126 Bari, Italy; 5 Hahn-Schickard, Institute for Microanalysis Systems, Sedanstrasse 14, 89077 Ulm, Germany

**Keywords:** chitosan antimicrobial, derivative TMC, biofilm inhibition, IR-ATR spectroscopy, molecular mechanisms, *L. parabuchneri* biofilms

## Abstract

Antimicrobial materials are considered potential alternatives to prevent the development of biofilm-associated contaminations. Concerns regarding synthetic preservatives necessitate the development of innovative and safe natural antimicrobials. In the present study, we discuss the in situ infrared attenuated total reflection spectroscopy (IR-ATR) investigations of the selective antimicrobial efficiency of chitosan in controlling the growth of *Lentilactobacillus parabuchneri* biofilms. The protonated charges of chitosan were additionally amplified by structural modification via methylation, yielding quaternized derivative TMC (i.e., N, N, N-trimethyl chitosan). To evaluate antimicrobial effectiveness against *L. parab*. biofilms, IR-ATR spectroscopy provided information on molecular mechanisms and insights into chemical changes during real-time biofilm inhibition studies. The integrated fiberoptic oxygen microsensors enabled monitoring oxygen (O_2_) concentration gradients within biofilms, thereby confirming the metabolic oxygen depletion dropping from 4.5 to 0.7 mg L^−1^. IR studies revealed strong electrostatic interactions between chitosan/its water-soluble derivative and bacteria, indicating that a few hours were sufficient to affect biofilm disruption. The significant decrease in the IR bands is related to the characteristic spectral information of amide I, II, III, nucleic acid, and extracellular polymeric matrix (EPS) produced by *L. parabuchneri* biofilms. Cell clusters of biofilms, microcolonies, and destabilization of the EPS matrix after the addition of biopolymers were visualized using optical microscopy. In addition, scanning electron microscopy (SEM) of biofilms grown on polystyrene and stainless-steel surfaces was used to examine morphological changes, indicating the disintegration of the biofilm matrix into individual cells. Quantification of the total biofilm formation correlated with the CV assay results, indicating cell death and lysis. The electrostatic interactions between chitosan and the bacterial cell wall typically occur between protonated amino groups and negatively charged phospholipids, which promote permeabilization. Biofilm growth inhibition was assessed by a viability assay for a period of 72 h and in the range of low MIC values (varying 0.01–2%). These results support the potential of chitosan and TMC for bacterial growth prevention of the foodborne contaminant *L. parabuchneri* in the dairy industry and for further implementation in food packaging.

## 1. Introduction

Microbial biofilms are communities of species of mixed populations covered by an extracellular polymeric matrix linked to various substrates [1,2,3]. In the food industry, biofilms cause highly contamination because of their intrinsic characteristics, allowing bacteria to bind strongly to a range of surfaces, including rubber, polypropylene, plastic, glass, stainless steel, and even food products within just a few minutes. Subsequently, mature biofilms develop within a few hours to few days [4,5]. A main challenge in a food processing environment is the fact that bacteria, along with other molecules such as proteins from milk, dairy products, and meat, are absorbed into the surface, forming a conditioning film that attracts more biomass attachment. Thus, organic and inorganic molecules are transported to the surface by diffusion or turbulent flow of the liquid, creating an increasingly robust bacterial biofilm [6].

Biofilms of foodborne bacteria from the genus lactic acid bacteria (LAB) are of particular concern, given their high potential for spoilage and resistance to environmental stress [7]. Several studies have shown that genes of LAB species are responsible for biofilm adhesion [8]. Among them, *Lentilactobacillus parabuchneri* isolated from cheese has been reported to cause histamine poisoning symptoms such as sweating, headaches, flushing, diarrhea, and vomiting [9,10]. Knowing that histamine is a heterocyclic amine synthesized from the microbial decarboxylation of the amino acid histidine, it poses high potential risks to human health as a matter of food safety due to microbial contamination [11]. Dairy histamine-producing species form biofilms with a pronounced contamination capacity [12,13]. *L. parabuchneri* is a Gram-positive, facultatively anaerobic bacterium known as an undesired species in fermented food (cheese and other dairy products) that has low oxygen but high lactic acid availability [9,14,15]. Contamination caused by robust biofilms is a substantial consequence in cheese processing industries, especially for the post-ripening processing (i.e., cutting, slicing, and grating) of different types of cheese [16,17]. Advanced strategies to combat biofilms include inhibition and efficient eradication of biofilms. With growing awareness and concerns regarding chemically synthesized preservatives, novel and safe natural antimicrobials that target food pathogens with minimal adverse effects have attracted attention. The objective of this study was to analyze the properties of chitosan as a natural antimicrobial agent for controlling the growth of *Lentilactobacillus parabuchneri* biofilms. To the best of our knowledge, this is the first time that the inhibition of the bacterial food contaminant *Lentilactobacillus parabuchneri* and its biofilms by chitosan and its derivatives has been studied at the molecular level using real-time IR-ATR spectroscopy. 

Chitosan, a deacetylated polysaccharide derived from chitin, is a biocompatible compound with pronounced inhibitory activity, and is especially suitable for addressing sessile communities of lactic acid bacteria. Chitosan films are regarded as bifunctional materials that are well tolerated by living tissues and are particularly applicable as edible coatings to prolong shelf life and preserve the quality of fresh foods. The wide use of chitosan, on account of its tunable properties and its anti-inflammatory and antimicrobial activity, makes chitosan and its synthesized derivatives a potential biomaterial for wound healing, dentistry [18] and, in particular, in the medical field, chitosan films have been tested as curative wound dressings and scaffolds for tissue and bone engineering [19]. Chitosan is used as an immunostimulant for the protection of fish from bacterial diseases and as a dietary supplement, but with a level of toxicity when dissolved in an acidic environment [20]. Currently, polymeric nano-biomaterials are being studied for the preparation of nanoscale drug carriers, considering the hazard assessment of chitosan for medical use [21]. Studies evaluating the antimicrobial activity of chitosan against different groups of microorganisms suggested that three main mechanisms of microbial growth inhibition are involved [22]. The proposed basic mechanism relies on the cationic amino groups of chitosan, which increase the permeability of the negatively charged outer cellular layer, causing the disruption and release of intracellular components [23,24,25]. Regarding the fact of being attractive biomolecules, chitosan-based antimicrobial applications are limited due to their water insolubility [19]. Water-soluble chitosan derivatives frequently exhibit enhanced antimicrobial activity compared with chitosan itself [26,27]. Therefore, owing to the lack of a positive charge at neutral pH and low solubility, a large number of chitosan derivatives have been developed by modification with amine and hydroxyl groups via carboxylation, alkylation, and quaternization [27,28,29].

The cationic polyelectrolyte characteristics of polymer chains with positive charges are enabled by quaternization of the nitrogen atoms of the amino groups. The alkyl moiety of the resulting N,N,N-trimethyl chitosan facilitated structural affinity between the derivative and the microbial cell wall [26,30]. The antimicrobial activity of chitosan and its methylated derivative has been observed for a variety of *Lactobacillus* species related to infections, contaminations, and mediated fermentation processes [31,32,33], and antimicrobial activity against foodborne pathogenic bacteria and spoilage microorganisms has been proposed [34]. The inhibited growth of Gram-positive *lactobacillus* via the antimicrobial activity of chitosan reveals significant potential to extend the shelf-life of food by inhibiting the growth of food spoilage microbes [23]. The influence of chitosan on the inhibition of lactic acid bacteria is dependent on the range of minimum inhibitory concentrations (MICs) [35] and the interaction mechanism within the bacterial cell wall. The main advantage of the methylated derivative of chitosan with quaternization of the amino groups is the generation of permanent positive charges on the polymer chains, which are available for ionic interactions with the negatively charged cell surface of the bacteria [36]. Chemically modified chitosan with desired physicochemical properties, including solubility and hydrophilicity, appears to provoke extracellular and intracellular effects [28,37,38]. The biological characterization of chitosan coatings assessed via cell viability and antimicrobial tests revealed the biocompatibility of osteoblasts, as well as antiadhesive and antibiofilm activity against both Gram-negative and Gram-positive bacterial strains [22,39,40].

Infrared attenuated total reflection spectroscopy (IR-ATR) is a powerful analytical technique that facilitates the tracking of molecular and metabolomic changes in bacteria and associated biofilms in real-time [41,42]. Therefore, in recent years, IR-ATR spectroscopy has gained attention for studying biofilms at the molecular level, given its capability for in situ monitoring of the chemical composition of biofilms in their hydrated state under various environmental conditions [15,43,44]. This enables both studies on the early stages of biofilm formation as well as bacterial attachment and colonization at the ATR waveguide surface [45,46]. The antibacterial, antibiofilm, and anti-adhesion properties of different compounds and their bioactivity can be periodically monitored against bacteria present within a biofilm [47,48]. As IR spectroscopy focuses on the detection of the major structural groups of the biofilm developed at different stages, one may be observed during actual biofilm formation [49,50,51]. IR-ATR spectroscopy is a highly suitable approach for obtaining long-term information on the temporal behavior of molecular biofilm constituents, chemical properties, and changes in the metabolic activity of surface-associated bacterial growth [52]. The absorbance intensity changes owing to the peak assignment of the individual absorption bands assigned predominantly to cell walls consisting of a rigid peptidoglycan framework, capsular lipids, fatty acid chains, membranes, ribosomes, and polysaccharides in cellular microbial communities [15,53]. Bacterial attachment to the bare ZnSe waveguide is associated with amide I, amide II, nucleic acids, amide III, and extracellular polymeric substances, which indicate biofilm growth and increase significantly over time [54]. Adhesion driven by van der Waals and electrostatic forces is responsible for the interaction of the surface with the host cells [55]. For long-term biofilm monitoring, the interaction with the surface is influenced by the addition of cationic antimicrobials. The electrostatic interaction of cationic chitosan with negatively charged bacterial surface layers is embedded in the hydrophobic regions of lipid membranes, thus causing membrane damage and disintegration [56,57]. As a result of bacterial cell damage, the IR intensity of all bands related to biofilms slightly decreased over time, indicating that less biofilm was attached to the waveguide surface.

In the present study, we aimed to investigate the potential of chitosan and its methylated derivatives as novel natural antimicrobials to inhibit *Lentilactobacillus parabuchneri* biofilms. We directly explored the effects of chitosan on the *Lentilactobacillus parabuchneri* DSM 5987 strain via IR-ATR spectroscopy at the molecular level. Microbiological tests confirmed the antimicrobial and antibiofilm activities of the chitosan and its methylated derivative, TMC. Furthermore, scanning electron microscopy (SEM) insights into bacterial cells suggest that the membrane permeability of the materials causes biofilm inhibition. 

## 2. Results

### 2.1. IR Characterization of Chitin and Chitosan

Prior to evaluating the antimicrobial efficacy of chitosan, characterization of commercially available chitosan via determination of the deacetylation degree (*DD*) as an important factor for substitution and increased inhibition effect was conducted [36,58]. *DD* is closely related to the antimicrobial effect of chitosan; the higher the value of *DD*, the more protonated the amino groups available for electrostatic interactions with the cell surface, provoking cell wall disruption and cell death [33]. Therefore, the growth of the biofilms formed by lactic acid bacteria is expected to be effectively inhibited.

The Brugnerotto equation [59] is as follows:

(1) $$\frac{A_{1320}}{A_{1420}}=0.3822+0.03133\;DA$$

(2) DD%=100−DA%

(3) $$A_{1320}=0.03206\;\;$$

(4) $$A_{1420}=0.0370$$

(5) Degree of deacetylation (DD)=78.86%

**Figure 1:**
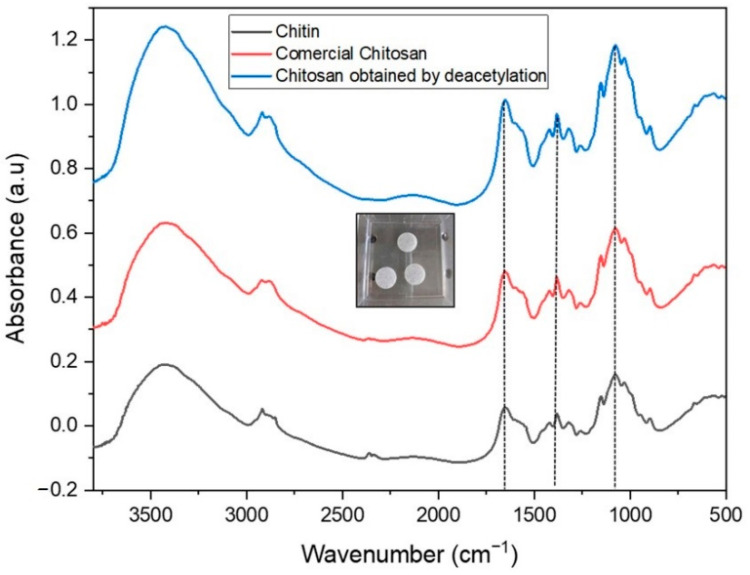
Transmission IR spectra (using the KBr pellet technique) of chitin (black spectrum), commercial chitosan (red spectrum), and deacetylated chitosan (blue spectrum) to determine the influence of *DD* on the efficiency of chitosan against bacterial biofilms.

Commercial chitosan showed variance in chitin source content. The deacetylation process was observed using transmission infrared (IR) spectroscopy. The chitosan sample (2 mg) was ground and mixed with 200 mg of KBr and compressed in a hydraulic press to obtain KBr pellets. IR spectra (see Figure 1) were recorded in the range 4000–500 cm^−1^, averaging 128 scans at a spectral resolution of 2 cm^−1^ using a Tensor II FTIR spectrometer in the transmission mode (Bruker Optik GmbH, Ettlingen, Germany).

The IR data revealed characteristic peaks of chitosan, in line with previous studies [33,60]. These include peaks at 1153 cm^−1^ (C−O−C stretching), bands at 2920 cm^−1^, 2886 cm^−1^ (CH_3_ and CH_2_ stretching), and 3443 cm^−1^ (−OH stretching), the band corresponding to amide I in the 1654 cm^−1^ region, which is related to the antiparallel alignment of αchitin, angular deformation of the N–H bond (amide II) at 1560 cm^−1^, and axial deformation of C–N (amide III) at 1321 cm^−1^. The band at 1383 cm^−1^ represents the acetamide group of the chitosan molecule. At 1153 cm^−1^, an axial symmetric deformation signature of C–O–C was evident [33]. The IR spectra of the commercial and deacetylated chitosan showed similar characteristics and were comparable to those reported in the literature. Commercial chitosan with a medium molecular weight (MW = 50,000−2,000,000 Da, Sigma Aldrich) had a deacetylation degree of 78.86%.

### 2.2. Biofilm Inhibition by Chitosan

The antimicrobial effectiveness of chitosan polysaccharide as a biofilm-destructive solution was confirmed by IR spectroscopy. The purpose of this study was to demonstrate the influence of chitosan on the molecular processes occurring in *Lentilactobacillus parabuchneri* DSMZ 5987 during biofilm formation and the associated inhibition of bacterial growth. Real-time monitoring via flow-through in situ IR biofilm analysis was performed until the mature biofilms were observed. First, a nutrient-rich conditioning film is formed. A stationary growth phase was observed after the introduction of the bacterial suspension. At this stage, the suspension contained approximately 1 × 10^9^ cells/mL, ensuring reproducible results.

**Figure 2:**
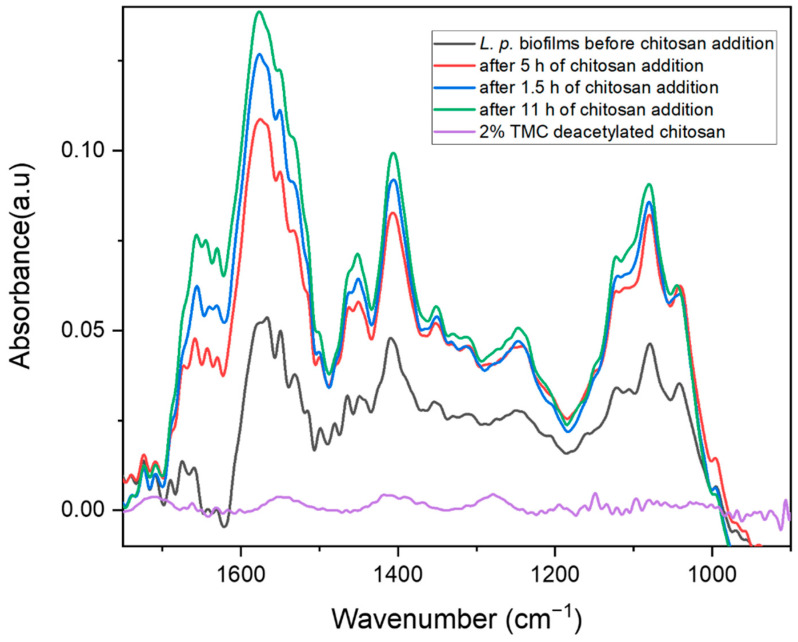
IR-ATR spectra of *L. parab*. biofilms with a 2% stock solution of chitosan in 1% acetic acid solution and MRS medium. The IR spectra were baseline-corrected.

The IR spectrum of chitosan (Figure 2) showed a significant decrease in the IR signature characteristics of biofilms within nearly the entire spectral range of 1650–900 cm^−1^ after the first hours of 2% chitosan insertion (i.e., amide I and II, lactate, and EPS spectral band reduction). The interaction between chitosan and the bacterial cell wall is mostly electrostatic, and typically occurs between protonated amino groups and negatively charged phospholipids, which promotes permeabilization. Hence, the chitosan solution exhibited pronounced antimicrobial activity against the bacterial contaminant *Lentilactobacillus parabuchneri*. The ability of this antimicrobial to selectively degrade the cell wall of microorganisms [39] significantly inhibits biofilm formation and reduces metabolic activity and the secretion of the EPS matric [61]. The prolonged bacteriostatic action of chitosan to inhibit the growth and reproduction of *L. parabuchneri* renders this cationic polymer capable of preventing biofilm formation and eradicating mature biofilms. The functionality of chitosan as an antimicrobial depends on the cell membrane of microorganisms due to ionic interactions, which alter the membrane permeability leading to cellular lysis [62,63]. Bacterial survival relies on complex multicellular responses via cell-to-cell communication via quorum sensing [15]. A better understanding of the dynamic lifecycle of *L. parabuchneri* and the surface adherence to biofilms influenced by chitosan insertion allows the examination of the link between microbial growth and survival. The temporal evaluation of *L. parabuchneri* biofilms is investigated via intensity changes of infrared bands over a period of four days (Figure 2).

**Figure 3:**
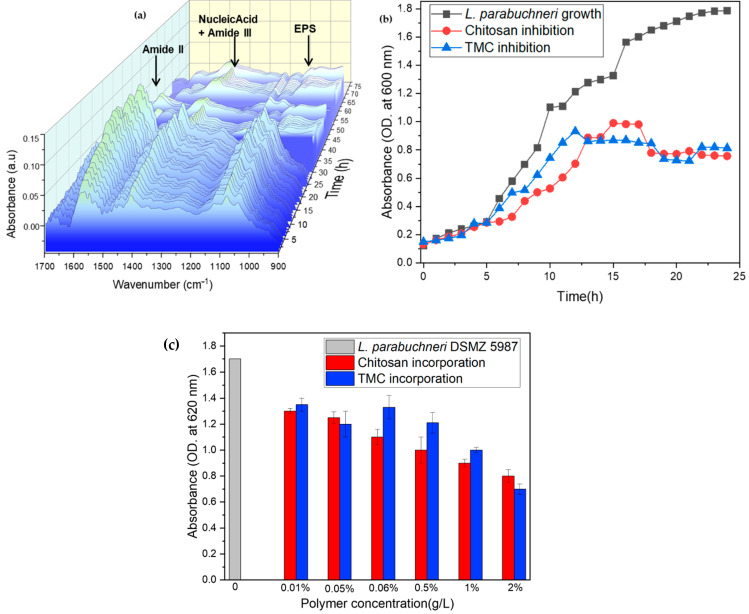
(**a**) Three-dimensional plot of IR-ATR spectra during three days of monitoring of temporal biofilm inhibition, (**b**) growth profile of *L. parabuchneri* DSMZ 5987 as assayed by turbidimetry at 620 nm compared to chitosan and TMC in 2 g/L concentration added to the MRS medium during a 24 h interaction with bacteria, and (**c**) inhibition histogram of chitosan and TMC derivative as a function of different polymer concentrations added to MRS media for 16 h of antimicrobial-biofilm interactions. The error bars represent the standard deviation of three independent experiments.

The appearance of decreased bands at 1646 and 1560 cm^−1^ is in accordance with major changes related to parallel and antiparallel β-sheets of the amide I and II bands, while the spectral region around 1402, 1374, and 1221 cm^−1^ is indicative of the reduction of carboxylate groups near the active surface of the ATR waveguide for both the amide II and III band regions [59]. Concerning the decreasing signals at 1272, 1221, and 1129 cm^−1^, which are related to the presence of nucleic acids and EPS, a decrease in peak height was apparent even after 1.5 h of inhibition with chitosan. Decreased phosphate diester, polysaccharide, and phospholipid peaks support the antimicrobial contribution of chitosan contained in the growth medium compared to that of *L. parabuchneri*. Decreasing bands correspond to β-sheet structures, helices, and asymmetric carboxylate groups, which may indicate bacterial biofilm degradation and cell detachment within the first hours of incubation with a 2% deacetylated chitosan solution [64]. Owing to the antimicrobial activity following chitosan addition, there was a significant decrease in the IR signal response within nearly the entire spectral range (i.e., 1700–900 cm^−1^) after 11 h of chitosan insertion (Figure 2). The temporal evolution of *L. parabuchneri* biofilm IR spectra monitored for three days with chitosan incorporation into MRS media is shown in Figure 3a. 

The initial phase of bacterial attachment and the associated biofilm growth indicated distinct disruptions. The biofilm matrix appeared to be depleted in nutrient sources, and less lactic acid, nucleic acid, and EPS were synthesized. During progression, the decreasing signals for β-turns (i.e., amide I), random coils (i.e., amide III), and phosphate diesters in the amide III band region signified inhibition of biofilm formation.

Throughout the measurement period, different adhesion kinetics [65] were observed, indicating potential changes in culture conditions, growth phase properties, and bacteria inoculated into the flow system. The growth kinetics of *L. parabuchneri*, as measured by turbidity at 620 nm, is shown in Figure 3b, where OD is the optical density. Bacteria grown in the absence of an antimicrobial showed an exponential increase in growth (i.e., log phase) during the first 6–8 h, followed by a stable stationary phase. The turbidity readings of the inoculated solutions after the addition of the polymer and its derivative were then carried out for 24 h of incubation, ensuring that the maximum number of microorganisms per volume (plateau) was present. Once the antimicrobials were mixed with the broth, the growth rate was temporarily affected, causing a rapid reduction in maximum absorbance. These results indicate that the addition of chitosan reduces bacterial proliferation in a linear manner, but with a short range of absorption intensity differences compared with the synthesized derivative known as trimethyl chitosan. The N, N, N-trimethylchitosan (TMC) derivative was also efficient in reducing the number of colonies compared with the control culture medium. A detailed study of growth colonies and reduction of Gram-positive *L. parabuchneri* contaminants influenced by the insertion of chitosan and TMC after 16 h of interaction with the microbial solution as a function of antimicrobial concentrations is shown in Figure 3c. The optical density histogram indicated that both chitosan and its methylated derivative anticipated a reduction in microbial proliferation, with small statistical differences. The biopolymer chitosan at concentrations of 1 and 2 g/L resulted in low absorbance intensities, whereas the TMC derivative was not concentration-dependent. For all tested polymer concentrations in the gel and liquid state (0.01–2.0 g/L), chitosan and its charged derivative TMC showed a similar effect against *L. parabuchneri* with an evident linear tendency in reducing bacterial population as the concentration increases.

### 2.3. Biofilm Inhibition by Methylated Chitosan Derivative (TMC)

**Figure 4:**
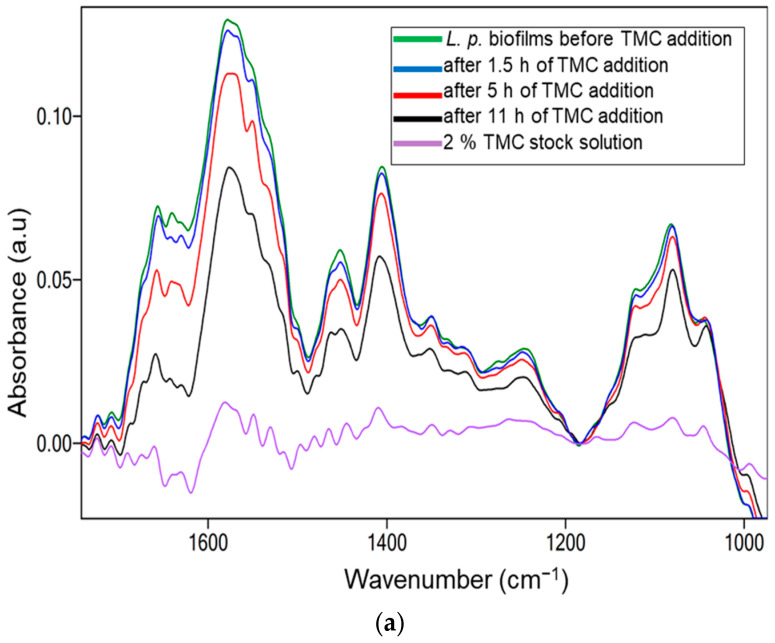

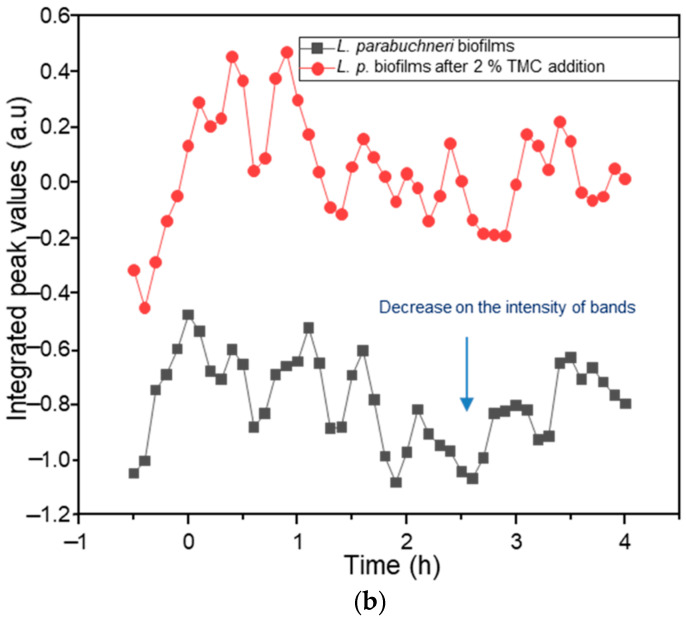
(**a**) IR-ATR spectrum of *L. parabuchneri* biofilm disruption after the insertion of TMC; (**b**) integrated peak values (IPVs) as a function of time for the spectral window 1700–900 cm^−1^.

Quaternization of chitosan is a chemical modification of this cationic polysaccharide by forming a water-soluble derivative known as N-quaternized chitosan [29,36,66]. The bioactivity of trimethyl chitosan against *L. parabuchneri* biofilms was evaluated following the same protocol as that used for the initial chitosan solution. However, enhanced antimicrobial activity was expected because TMC has a permanent positive charge on its polymer backbone. The accumulation of biomass at the ATR crystal surface and the decrease in bacterial coverage can be explained by the related IR absorption features [54]. IR-ATR spectra of *Lentilactobacillus parabuchneri* biofilms were monitored for three days after the addition of TMC, showing a decrease in spectral signatures corresponding to proteins (Amide I, Amide II), lactate, amide III, nucleic acids, and polysaccharides. The spectral region 1700–900 cm^−1^ known as the representative region for the identification of *L. parabuchneri* biofilms, enables the investigation of inhibitory mechanisms during the period of controlled biofilm growth. A similar decrease was observed with chitosan, indicating a reduction in the corresponding band intensities after the addition of 2% TMC (see Figure 4a).

Changes in integrated peak areas of spectral signature characteristics of biofilms, that is, amide I and II (1700–1464 cm^−1^), amide III (1350–1200 cm^−1^), nucleic acids (1280–1180 cm^−1^), and extracellular polymeric substances (1121–997 cm^−1^), are shown with a slight periodical decrease in the IR signals (Figure 4b). A significant decrease in the biofilm level was observed after only 2 h of inoculation of the TMC solution into the IR-ATR setup. Considering the viscosity of the aqueous TMC solution introduced into the flow system, the surface of the mature biofilm directly formed at the ATR waveguide slowly disintegrated by this natural antimicrobial. The IR band centered at 1457 cm^−1^ is related to asymmetric C-H stretching in the methyl groups and is more evident in the derivative spectrum. The most significant reduction was apparent in the extracellular polymeric matrix signals, indicating the inhibition efficiency of the polysaccharides, nucleic acids, and phospholipids (Figure 4b).

The optical density of the bacterial suspension introduced into the IR-ATR flow system was OD_600_ = 1.2. The OD value of the suspension containing TMC after the measurements were stopped was OD_600_ = 0.7. The continuous inhibition of biofilm growth, monitored by turbidimetry measurements, proved that the positive charges of TMC enhanced the antimicrobial properties of chitosan (Figure 3b,c).

### 2.4. Monitoring Oxygen Concentrations during Biofilm Inhibition

Owing to the heterogeneous nature of biofilms, the spatial and temporal dynamics of additional physicochemical parameters such as oxygen levels are of interest. The evaluation of *L. parabuchneri* biofilms initially attached to the waveguide surface is closely related to metabolic changes inside nascent biofilms. The gradient of nutrients and associated oxygen consumption led to differences in the physiological state of the bacterial species in close proximity to the support material within the biofilms. Hence, changes in oxygen concentration during the first 12 h of the experiment complemented the simultaneously recorded IR data to improve the interpretation of the processes occurring during biofilm formation. 

The fluorescence-based optical sensor spot (PtTFPP) was immobilized on the ATR crystal between two active hotspots at the waveguide surface, resulting from total internal reflections [67]. This enables oxygen level measurements at the waveguide surface and within the biofilm without interfering with IR signals. A fiberoptic oxygen meter (Firesting, Pyroscience, Aachen, Germany) was used for this purpose [68,69]. The growth of the initial biofilm was influenced by the limited availability of dissolved oxygen. Being a facultative anaerobic bacterium, *Lentilactobacillus parabuchneri* requires microaerophilic conditions for its growth. This explains the continuous decrease in dissolved oxygen during the in situ monitoring of biofilm growth and inhibition.

**Figure 5:**
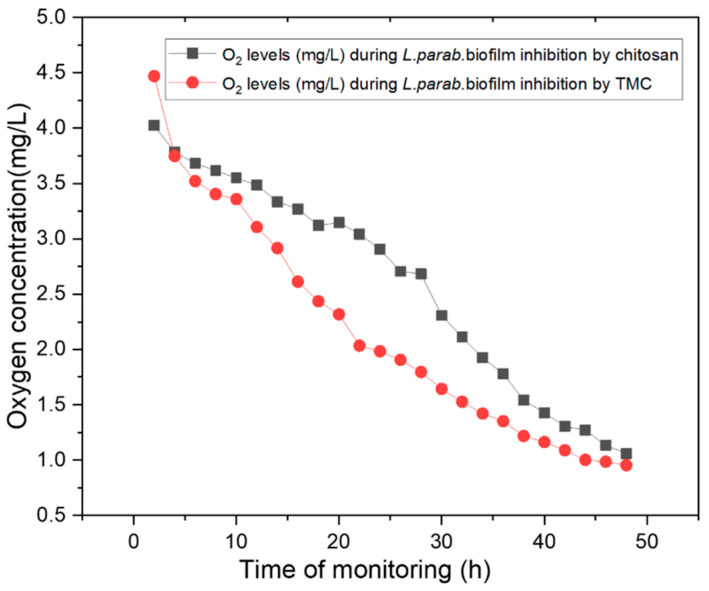
Concentration profiles for oxygen during bacterial detachment at the ATR waveguide surface at a constant flow of chitosan and TMC inhibition were determined via an oxygen optical sensor spot attached to the ATR waveguide surface. The results shown are the averages of three replicative oxygen concentration measurements.

The low oxygen consumption rate of *L. parabuchneri* DSMZ 5987 is caused by the microaerophilic conditions required inside the IR-ATR flow cell; thus, the oxygen concentration tends to reach an asymptotical value (Figure 5). The significant decrease in the oxygen consumption rate is evidence of the behavior of this facultative anaerobic bacterium in the regulation of anaerobic metabolism, as well as a proof of concept for maintaining microaerophilic conditions [70]. The decrement from 4.5 to 0.7 mg L^−1^, determined by the optical oxygen sensor, indicates the low metabolic oxygen present during biofilm formation as lactic acid is abundantly produced (see Table S1 at supplementary material). In situ oxygen measurements enable an understanding of the metabolism of this facultative anaerobic bacterium related to proliferation ability, changes in cellular composition, and the metabolomic cell response after exposure to chitosan and TMC antimicrobials [9,41]. Oxygen levels may change rapidly depending on the thickness of the biofilm [68,71]. Real-time measurement of the oxygen content on top of the ATR surface via MRS media exchange and the addition of chitosan and TMC as inhibitors of biofilms promoted biomass detachment. The metabolic stress response varied considerably between the different tested concentrations of chitosan and its derivative trimethyl chitosan towards biofilm inhibition and cell metabolic activity. A decrease in oxygen levels occurs naturally via bacterial respiration. Oxygen consumption in the cell membrane is regulated by terminal oxidases in the form of respiratory protection that allows anaerobic metabolism to function under microaerobic conditions [72,73]. Because of the influence of bioactive chitosan and trimethyl chitosan, cellular oxygen consumption in a microaerophilic environment [68] can be correlated to microbial growth inhibition via cell lysis [74].

Figure 5 shows the concentration profiles of oxygen during biofilm inhibition by chitosan and its methylated derivatives. Oxygen levels are dependent on biofilm disruption and dispersal of detached cells. The reproduction of microorganisms, which is disturbed by the introduction of natural antimicrobials, is associated with a reduction in oxygen levels at the base of *L. parabuchneri* biofilms (i.e., close to the ATR waveguide surface). The decrease in the oxygen value was attributed to the increase in biofilm thickness, resulting in a concentration gradient throughout the film [75]. The attachment of bacterial cells also occurs on the immobilized fiber optical sensor spot, providing real-time measurement of the oxygen concentration on the top of the surface under local anaerobic conditions.

Oxygen limitation is an important factor in the tolerance of *L. parabuchneri* biofilms to bacteriostatic inhibition by chitosan and TMC; however, it is apparently not the only factor. The formation of biogenic amines by decarboxylation of amino acids may be responsible for neutralization of the cellular environment by lactate metabolism [76]. Similarly, monitoring pH changes before and after biofilm formation revealed a decrease in pH, which was linked to histamine decarboxylation. Histamine production during biofilm formation is less pronounced in acidic cheeses [76]. A low extracellular pH increases survival under acidic stress. The analysis of pH in cellular microenvironments is representative of cell growth, proliferation, and metabolic activity. Cellular metabolism rates change over time towards lower pH values. In most cases, the extracellular pH becomes more acidic after flow-through monitoring than the initial neutral pH of the broth medium [76,77,78]. The pH value measured before chitosan incorporation was 5.8. After the biofilm inhibition, the pH of the system was reduced to 5.0. The TMC derivative with an additional methylated group prepared in a gel-neutral medium gave chitosan a cationic character, independent of the surrounding pH [65,79]. The lower values of interacting pH in the range 4.5–5.0 for the TMC derivative indicate that intense ion interaction reports on the ion donation initiating the hydrolysis of the peptidoglycan microorganism wall constituents [80,81]. 

In summary, IR-ATR and integrated co-localized oxygen sensing simplify biofilm studies in molecular detail, enabling unprecedented insight into antimicrobial activity and associated cell metabolic activities, facilitating temporally resolved monitoring of inhibitory activity.

### 2.5. Modeling EPS Production during Biofilm Formation and Inhibition

Using a cumulative impulse fit procedure based on individually recorded and modeled IR spectra of biofilms facilitated the evaluation of the respective contributions to the entire sum spectrum for visualizing the effect of chitosan and TMC on the EPS of *L. parabuchneri*. In addition to an apparent decrease in the spectral signature associated with EPS, a decrease in the spectral region dedicated to polysaccharides 1160–950 cm^−1^ is likewise evident. The calculated values for the investigation of the EPS-relevant peaks from the cumulative impulse fit of the spectra were monitored after 168 h of *Lentilactobacillus parabuchneri* incubation in MRS media and after 2.5 h and 6 h of chitosan and TMC addition.

Further expansion of the chitosan incubation resulted in a constant decrease of all signals in the EPS spectral window, which was reached once chitosan was added after the first hour of bacterial growth. This implies that there was reduced bacterial growth and a significant decrease in EPS production for the MIC concentrations of chitosan evaluated during real-time IR monitoring. Bacterial biofilm degradation and cell detachment became visible within the first hours of incubation with a 0.05 g/L chitosan stock solution. The intensity of the polysaccharide bands also decreased in the IR region of 1108.3 cm^−1^ and with 0.05 g/L TMC solution. Considering that the TMC concentration used has been shown to decrease bacterial biofilm formation and EPS production for other bacteria but does not completely inhibit bacterial growth, it is more likely that there are parallel defense and reorganization processes in the microbial biofilm, such as the production of antibiotic-degrading enzymes [82].

The strongest signals for *L. parabuchneri* bacterial growth inhibition were found in the 1150–950 cm^−1^ region marked by biofilms. In this spectral IR range, feedback of C–OH (alcohol), C–C, and C–H vibrations in a broad band is expected. Spectral modification of these bands at approximately 1070 cm^−1^, 1040 cm^−1^, and 990 cm^−1^ is typical of IR-ATR spectra of bacteria [83,84,85]. However, peak attribution in this spectral region (Table 1) remains difficult because several features of the functional groups overlap significantly. The increase in polysaccharide bands, which is related to EPS production during the bacterial biofilm maturation process, has been attributed to these spectral changes [86]. In addition, phosphodiester and polyphosphate products have been associated with the peak at approximately 1070 cm^−1^ and the symmetric stretching vibration of phosphoryl groups at around 990 cm^−1^ [83]. Phosphodiesters as key components indicate the maturation of biofilms; therefore, EPS production and *L. parabuchneri* mature biofilms were visualized under the influence of chitosan and TMC as antimicrobial agents.

**Figure 6:**
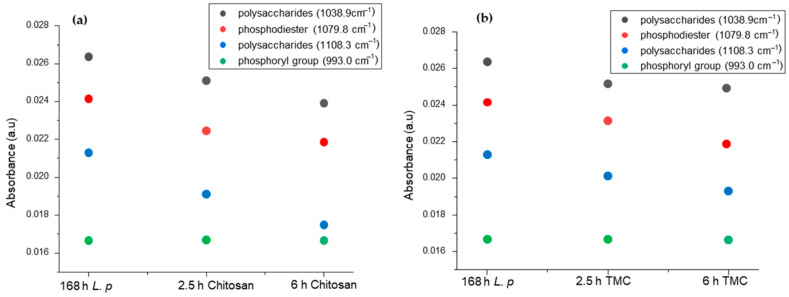
Calculated values between 1150–950 cm^−1^ for the band of polysaccharides (1038 cm^−1^, black, left), polysaccharides (1108.3 cm^−1^, blue, left), phosphodiesters (1079.8 cm^−1^, red, left), and phosphoryl group (993.0 cm^−1^, green, left), from the cumulative impulse fit of the IR spectra monitored after 168 h of *L. parabuchneri* incubation in MRS media and after 2.5 h and 6 h of (**a**) chitosan and (**b**) TMC insertion.

EPS are the molecular structures responsible for the relevant adhesion processes [22]. According to the cumulative impulse fitting strategy, the absorption intensities of the four selected peaks decreased substantially with time. As evidenced by the EPS matrix, the extracellular polymeric content produced during the early stages of *L. parabuchneri* biofilm formation was disrupted by chitosan and TMC, as seen in the IR-ATR spectral features. In Figure 6a,b, the spread of polysaccharides, phosphodiesters, and phosphoryl groups indicates differences in the disruption of biofilms by decreasing IR signals related to EPS matrices. Trimethyl chitosan showed a periodic decrease over time after the addition of aqueous TMC solution in a 7-day continuous flow system for *L. parabuchneri* biofilm monitoring. Phosphoryl groups showed a constant absorbance intensity even after 6 h of TMC and chitosan addition. In the case of the chitosan stock solution, a drastic decrease in the polysaccharide region and phosphodiesters was observed, confirming the disruption of cell walls in the biofilm microbial communities. To further elucidate the influence of the respective secondary structures on reversible or irreversible adhesion processes, future observations with varying concentrations of chitosan and TMC are promising to provide deeper insight into the molecular processes involved in the IR signature of *L. parabuchneri* biofilms. The observation of the spectral region from 1150–950 cm^−1^ demonstrates the exploration of relevant changes in the IR-ATR spectrum with the inhibition of the *L. parabuchneri* biofilm by the addition of a biofilm-degrading agent, chitosan, or its synthesized trimethylated derivative. The influence of the antibacterial effects of chitosan and its quaternized derivative TMC on molecular mechanistic changes and biofilm formation by IR-ATR spectroscopy has not been elaborated.

### 2.6. Multivariate Analysis of the Obtained IR Spectra

FTIR spectroscopy coupled with multivariate statistical analysis can identify and discriminate the molecular components that change during different phases of bacterial growth, as reflected by IR absorption datasets [87,88]. Attenuated total reflection vibrational spectroscopy combined with chemometric models provides precise information on the chemical composition of surface-attached biofilms. This technique can be used to probe the internal changes caused by growth conditions and aging of microbial communities.

Principal Component Analysis (PCA) is widely used to discriminate the antibiotic agents related to the spectral differences between them by detecting antibiotic-treated biofilms based on changes in biochemical structures in real-time monitoring [89]. Orthogonal transformation, which finds a new set of variables retaining most of the sample information, tends to reduce the dimensionality of the datasets (sample). The so-called new variables or principal components (PCs) are fixed by the fraction of the total information contained in each variable.

**Figure 7:**
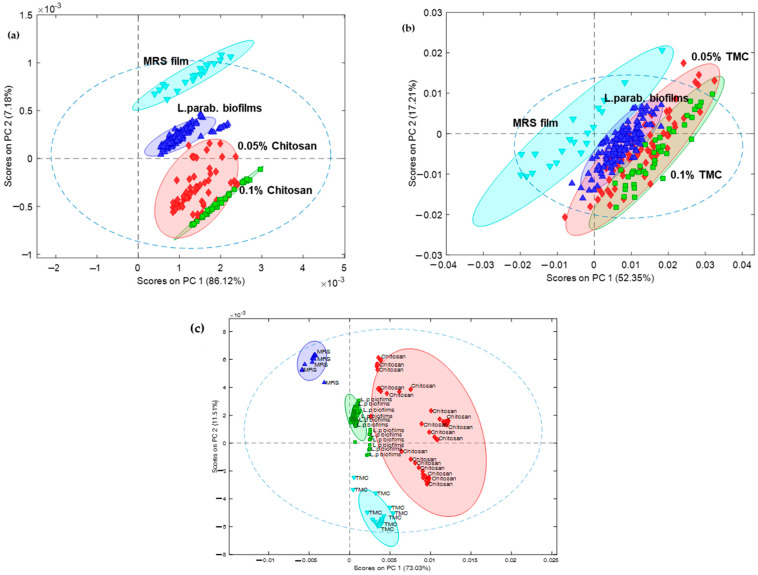
Multivariate data strategy for identification of the natural antibacterial performance of chitosan and its methylated derivative against *Lentilactobacillus parabuchneri* biofilms. (**a**) score plot of two PCAs of control biofilms treated with 0.05% and 0.1% chitosan, (**b**) PCA1-PCA2 plot of biofilm inhibition by 0.05% and 0.1% trimethyl chitosan (TMC), and (**c**) discriminant model based on principal component scores of real-time monitoring of reduced biofilms resulting from treatment with chitosan and TMC.

PCA decomposes the data matrix towards detection or finding “patterns”. The number of score values yields information on the spatial distribution of the chemical composition. The score maps for PCs provide insights into the performance of samples relabeled to the features [90,91,92]. The 2D scatter plot of the scores (Figure 7) illustrates the variation in the individual spectral windows for *Lentilactobacillus parabuchneri* biofilms influenced by chitosan (Figure 7a) and TMC addition (Figure 7b) without spatial information, where the two chosen principal components are the *x*- and *y*-axes. The PC1 versus PC2 score plots were generated from principal component analysis of the pre-processed data.

Measurement data of four-day old *Lentilactobacillus parabuchneri* biofilms linked to spectra recorded after 24, 48, 72, and 96 h of incubation with 0.05% chitosan and 0.05% TMC solution (MIC value) were thoroughly evaluated for their inhibition efficiency. PCA showed slight differences due to the changes in the inhibition performance of the inoculated strain and the measurement mode, while clusters were grouped into four different categories. Thus, spectral datasets of chitosan inhibition were best classified and explained by 2PCA at 93.3%, while the datasets from the TMC derivative addition were classified within 69.56% (Figure S1). In the case of the PCA performance for the comparison of chitosan and TMC together in one model, the best separation was observed (Figure 7c). The antimicrobial chitosan was time-sorted within the *L. parabuchneri* biofilms, whereas TMC was clustered separately in the model.

The spectral features represented by PCA1 and PCA2 scores in the antimicrobial active system for *Lentilactobacillus parabuchneri* biofilms highlighted the main chemical variance, mainly due to biofilm growth inhibition by the 0.05% chitosan stock solution. Biofilms disrupted over time indicated a change in the IR signature assigned for the first system, while some variation of chemical components related to biofilms after the addition of 0.05% TMC derivative solution was not influenced by the density of biofilm growth; therefore, the variability of biofilm biomass was not clearly addressed. The PCA model with its variance between relevant groups distinguished the biochemical and cellular structural changes induced by the natural antimicrobial agent chitosan and its synthesized trimethylated derivative.

### 2.7. Microscopic Studies of Biofilm Disruption

**Figure 8:**
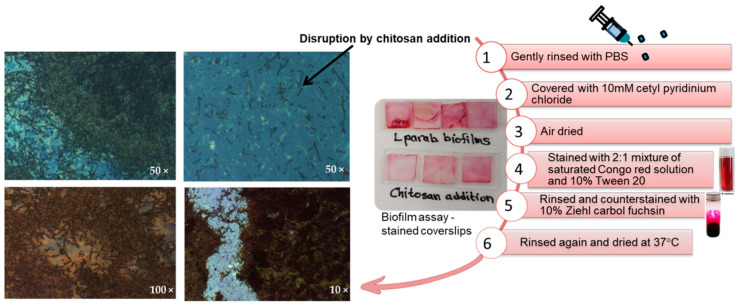
Optical micrograms of *L. parabuchneri* Gram staining. Congo Red-stained *L. parabuchneri* biofilms were grown for 10 days on glass coverslips and inhibited by the addition of a 0.05% chitosan stock solution.

Optical microscopy is an essential technique for studying bacterial biofilms and their surface topographies. Changes in biofilm parameters quantified by light microscopy and computer-controlled image analysis can be easily related to the surface area-averaged chemical behavior at the biofilm-substratum interface [93,94]. Biofilm maturity and disintegration processes were rated according to the presence of key structural features [95]. *L. parabuchneri* DSMZ 5987 biofilms formed over 10 days showed mature and homogeneous coverage on coverslip surfaces. Stained samples pretreated with 0.05% chitosan stock solution showed changes in the architecture of mature biofilms (Figure 8). Disintegration of the flat, homogenous cell layers of biofilms to the cell clusters and microcolonies, and destabilization of the amorphous polymeric extracellular matrix were observed to be different from the dense biofilms visualized in Figure 8.

In these biofilms, continuous layers of cells covering the coverslips with cluster-forming prominences were inhibited by chitosan. Optical micrograms of *L. parabuchneri* biofilms stained with Congo red, grown for 10 days on glass coverslips, and inhibited by the addition of 0.05% chitosan stock solution, indicated that major disruption occurred. The activity of chitosan, including this antimicrobial test, demonstrated inhibition of biofilm formation in the dispersion of the established mature biofilm. Thus, by means of the optical staining method, the structural form of biofilms can be understood by the heterogeneous destruction of the biofilm matrix.

**Figure 9:**
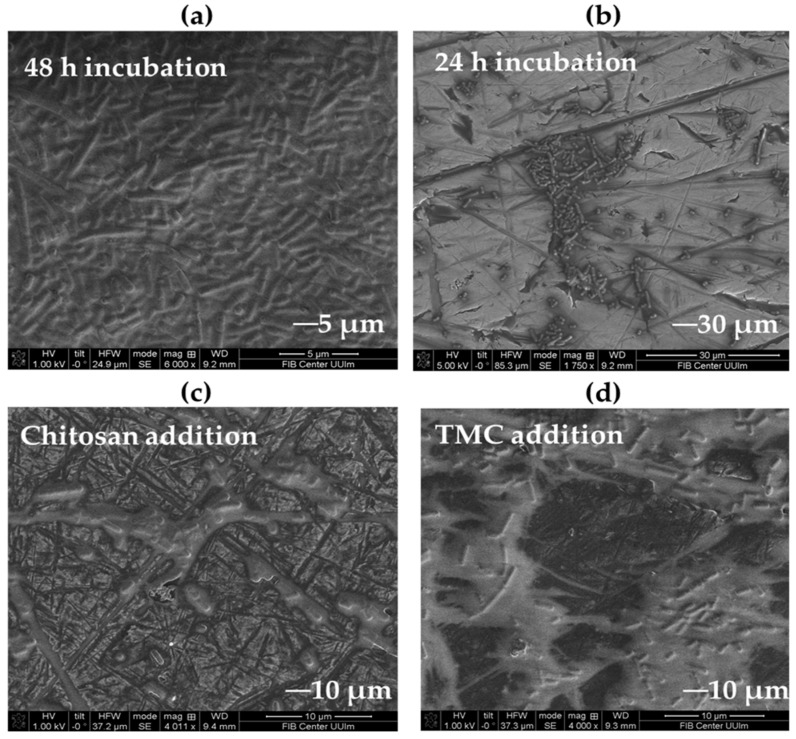
SEM micrographs of *L. parabuchneri* biofilm inhibition by chitosan and TMC; (**a**) after 48 h of incubation, (**b**) after 24 h of incubation, (**c**) after chitosan addition and (**d**) after TMC addition.

*Lentilactobacillus* biofilms can be formed on stainless steel, a common surface in the food processing industry [12,96]. The morphologies of the resulting biofilms and their inhibition efficiencies were observed by scanning electron microscopy (SEM). Figure 9a,b show SEM photomicrographs of the biofilm formed by *L. parabuchneri* DSM 5987 on stainless-steel coupons taken after 24, 48, and 72 h of incubation. A very uniform surface with dense structures of biofilms, clusters of cells embedded in the EPS matrix, and adhered to the coupons was visualized on top of the stainless-steel surface, which revealed an extensive first layer and well-defined *L. parabuchneri* homogenous biofilms. Patterns of the old biofilms on the substrate were supported by surface layer communities that were not regularly grown, knowing that the regions surrounding the edges of the biofilms were entirely filled with cells. A change in biofilm morphology was observed after the addition of 0.05% chitosan stock solution as a natural antimicrobial (Figure 9c) and its methylated derivative, TMC (Figure 9d). The antimicrobial activity of chitosan and TMC damaged the integrity of the microbial membrane.

In the samples incubated with natural antimicrobials, a decrease in the rate of substrate metabolism [97,98], changes in the bacterial community structure due to disintegration of the populations into individual cells, and microcolony destabilization were observed. 

The enhanced adhesion and high development rate of *L. parabuchneri* biofilms on bare stainless-steel surfaces reflect the architecture of a mature biofilm. After incubation for the first 6 h in media with chitosan/TMC addition, fewer cell aggregates grew, destroying the network of EPS matrices [99]. The bacterial cells quickly adapted to environmental changes, and a decrease in biofilm formation was observed after the first hour of incubation. The disintegration of the biofilm matrix into individual cells was due to the effectiveness of inhibition by chitosan and its quaternized derivative, as demonstrated by the separation of bacterial cells on the stainless-steel surface (Figure S3a,b).

### 2.8. Viability Assay—An Antibiofilm Study

The antimicrobial efficiencies of chitosan and its methylated derivatives on biofilm formation and inhibition of polystyrene surfaces were tested. A microtiter plate test assay for quantification of biofilm production was performed in triplicate [100]. Because this is a quantitative assay method, there is high accuracy of results on biofilm inhibition [101]. The results of the assay test with chitosan and TMC addition were given as a percentage of biofilm inhibition using the following formula:

(6) $$\%\;\mathrm{Biofilm}\;\mathrm{formation}\;\mathrm{inhibition}=100-({\mathrm{OD}}_{\mathrm{assay}}/{\mathrm{OD}}_{\mathrm{control}})\times 100$$

Biofilm formation on microtiter plates had OD_600_ = 2.38 ± 0.2 after 48 h of incubation and OD_600_ = 2.93 ± 0.5, after 72 h of incubation. A staining test for visualizing biofilms after the addition of chitosan and TMC at sub-MIC concentrations [101] was performed following the same step. A significant decrease in the optical density value to 1.46 ± 0.5 was found to produce less biofilm grown inside the system, proving that the percentage of biofilm formation inhibition [100,102] is 50.1% ± 0.7, while, for TMC, the optical density of 1.59 ± 0.1 brings about 45.7% ± 0.5 of biofilm inhibition efficiency:

(7) $${\mathrm{OD}}_{\mathrm{assay}}=1.46\;\mathrm{Chitosan}$$

(8) $${\mathrm{OD}}_{\mathrm{assay}}=1.59\;\mathrm{TMC}$$

(9) $${\mathrm{OD}}_{\mathrm{control}}=2.93\;$$

(10) % Biofilm formation inhibition=50.1% by Chitosan;45.7% by TMC 

**Figure 10:**
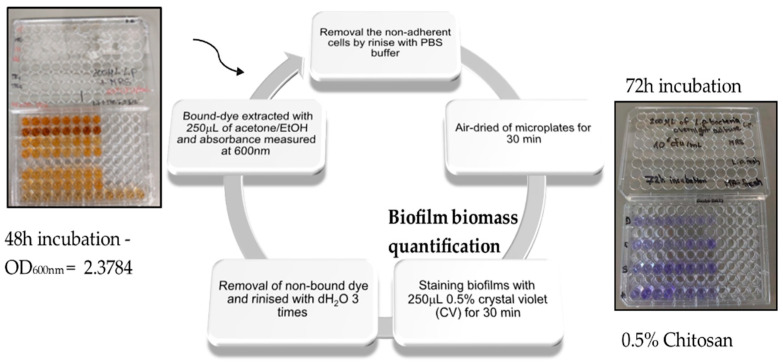
Overview of biofilm inhibition and quantification by crystal violet staining of polystyrene microtiter plates after 24, 48, and 72 h. The data represent the average of three biological replicates of the biofilm formation CV assay.

The utilization of chitosan and its methylated derivative in the prevention of *L. parabuchneri* biofilms with the capability to inhibit the formation of strong biofilms has proved to be the optimal choice for antibiofilm studies. Figure 10 shows the 96-well microtiter plate of the crystal violet (CV) assay to evaluate the inhibitory activity of chitosan and TMC. Quantification of total biofilm formation correlates with CV-stainable material and is better explained by cell death and lysis [103]. Multicellular biofilms with their developed microcolonies were subpopulated with viable cells [104]. In the case of biofilm inhibition, there is a disruption of cells associated with biofilm reduction (damage). The few microcolonies present that communicate via cell-cell signaling are coordinated to biofilm development and dispersal. On the other hand, cell death is a normal component of the development of multicellular communities. The death of individual bacterial cells contributes to the increased survival of the population [105]. Less than one percent of microbial cells undergoes lysis during biofilm development proving that this small fraction provides sufficient cellular DNA for biofilm stability [106]. This stability is disrupted by the biofilm inhibition mechanism of chitosan and its derivative TMC. Plasma membrane rupture and subsequent loss of intracellular content cause cell death and efficient microbial reduction. The important physiological process of cell death inside microcolonies is the key phenomenon resulting in the subpopulation dispersal of surviving biofilm cells still attached to the microtiter plate (Figure 10).

Chitosan exhibits intrinsic antimicrobial activity against the food-contaminating bacteria *L. parabuchneri*. This is because the biological mechanism of chitosan in interaction with microbial biomass promotes structural changes in the membrane wall leading to the impairment of surface cell structures and bacterial death [107]. The growth was obviously reduced to 50% after chitosan addition to the flow IR-ATR system. The minimum inhibitory concentration (MIC) of chitosan used for the viability assay was 0.01%. Bioactivity analysis showed that, for *L. parabuchneri* biofilms, chitosan had a superior percentage of inhibition and, thus, a higher ability to inhibit the adherence of bacteria compared to the TMC derivative, which offers a slightly lower inhibition. Minimum inhibitory concentrations (MICs) were determined using microdilution antimicrobial susceptibility tests [108]. The minimum concentration that inhibits the growth of pathogens through overnight incubation is traditionally derived from serial two-fold dilutions. The MIC values tested (0.01%, 0.05%, 0.06%, 0.1%, 0.5%, and 1%, taken from the 2% chitosan stock solution and TMC) showed no significant differences in the increment of inhibition performance. The inhibition activity with the highest inhibition value was observed after 72 h of incubation for 0.5% chitosan stock solution, whereas, for N,N,N-trimethyl chitosan (TMC), similar behavior of inhibition was observed in the range of concentrations 0.1–0.5% under all the tested conditions. The 72 h assay was taken into consideration as it offers realistic values of inhibition percentage for both systems. The capacity of chitosan to inhibit *L. parabuchneri* biofilms appeared to be concentration-independent, with high inhibitory activity even at the lowest concentrations. Naturally abundant chitosan and its methylated derivatives could be appealing to the dairy product industry for safe packaging, as they can effectively inhibit the growth of biofilms and prevent food-related contamination.

## 3. Materials and Methods

### 3.1. Bacterial Cultivation

*The Lentilactobacillus parabuchneri* DSM 5987 strain was used to evaluate the antimicrobial efficiency of chitosan and its derivatives. The lactic acid bacterial strain was obtained from the Leibniz Institute, German Collection of Microorganisms and Cell Cultures (DSMZ), Niedersachsen, Germany. To assess biofilm formation, *L. parabuchneri* was cultivated in Mann de Rogosa-MRS broth at 30 °C for 24–48 h. As this bacterium requires microaerophilic conditions, the inoculated Petri dishes were hermetically packed with anaerobic compact paper and incubated using the same cultivation steps. The supernatant was discarded and resuspended in fresh MRS medium, stirred for 2 h, and the OD was measured. The cells were resuspended in fresh MRS medium to reach an OD_600_ of 0.7, ready for in situ IR spectroscopy. Before inoculation into the flow system, the media was flushed with nitrogen gas (degassing process), and to create a microaerophilic environment for constant biofilm growth, the medium was degassed, and the levels of oxygen were measured using an Oxygen Retractable microsensor. The strain was isolated at −80 °C in MRS with 10% (*w*/*v*) sterile glycerol.

### 3.2. Stock Solution and Methylation Process

The chitosan polymer with medium molecular weight used for the studies was purchased from Sigma Aldrich (St. Louis, MO, USA). A deacetylation degree of 75–85% and molecular weight of 400,000 g/mol were the characteristics of the present deacetylated chitin (poly (D-glucosamine)). A 2% *w*/*v* chitosan solution in 1% acetic acid was used as the stabilizing and reducing agent during the initial nucleation stage. In addition, 2% (*w*/*v*) chitosan stock solution was prepared by dissolving 2 g of chitosan in 1% (*v*/*v*) acetic acid to obtain a total volume of 100 mL, and this solution was stirred under constant heating (60 ± 5 °C) for 24 h at an ambient temperature. Similarly, chitosan solutions at 0.01%, 0.05%, 0.06%, 0.1%, 0.5%, and 1% (*w*/*v*) concentrations were obtained.

**Figure 11:**
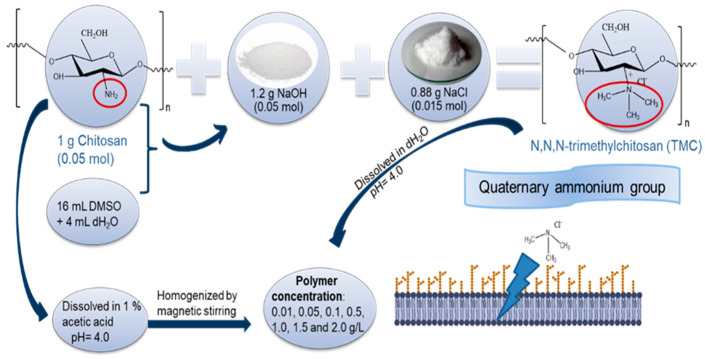
A schematic representation of the synthesis steps for the methylation process of the quaternization of the amino groups of chitosan resulted in N,N,N-trimethyl chitosan (TMC).

The methylation process was based on methods described by Goy et al. [65] and de Britto et al. [36]. Briefly, the reaction consists of the addition of 1.2 g of 15 mM NaOH and 0.88 g of 15 mM NaCl in a suspension of 1 g of 5 mM chitosan in 16 mL of dimethyl sulfate (Synth, R. Janeiro, Brazil) and 4 mL of deionized water. The mixture from the methylation process was stirred, and the quaternate derivative N,N,N-trimethyl chitosan (TMC) obtained by precipitation with acetone was rinsed and air-dried. The characteristic feature of the new derivative is that methyl groups are inserted at the C-2 position of the chitosan amino groups. Figure 11 shows the synthesis scheme of the quaternized chitosan derivative.

To investigate the differences in the degree of deacetylation, commercial and naturally deacetylated chitosan were used for comparison. Stock solutions of chitosan and its methylated derivatives for inhibition analysis were prepared in 2% acetic acid (pH = 5–6). After stirring for 1 h, polymers with different concentrations were prepared.

### 3.3. Vial Cell Assay

The ability of each strain to form a biofilm on polystyrene was tested using overnight cultures (MRS broth) diluted to approximately 10^6^ CFU/mL. Eight wells of a round-bottomed polystyrene 96-well microtiter plate were inoculated with 200 µL of each dilution (in duplicate). The negative control consisted of eight wells filled with sterile medium. The plates were then incubated at 30 °C. Biofilm biomass was quantified using the crystal violet staining method (CV assay) described here, with modifications [7]. After 20, 24, 28, 32, 48, and 72 h of incubation, the supernatant was removed, and all wells were rinsed twice with PBS to remove non-adherent cells. The plates were then air-dried for 30 min at room temperature. Any biofilm present was then stained with 250 µL of 0.1% (*w*/*v*) crystal violet in distilled water (dH_2_O) for 30 min at room temperature (4 wells were untouched due to the inhibition test). The unbound dye was removed, and the sample was rinsed three times with 300 mL of dH_2_O. Finally, the bound dye was extracted using 250 mL of acetone/ethanol (80/20) and the absorbance was measured at 600 nm using a UV-Vis spectrometer (Specord S600, Analytik Jena AG, Jena, Germany).

### 3.4. Biofilms Grown on Glass Coverslips

The simplest biofilm architecture was visualized using light microscopy. The biofilm assay and staining method were performed with some modifications [95]. Briefly, 300 µL of MRS medium were added to a sterile 13 mm coverslip in a well of a 24-well microtiter plate for the biofilm assay. The same protocol is followed for samples pretreated with 0.05% chitosan stock solution. Triplicate microtiter plates (technical replicates) were prepared and incubated under anaerobic conditions at 30 °C. The coverslips were incubated within the microtiter plates and left undisturbed for 10 days to mimic the average period of mature biofilm development. Upon removal, the coverslips were gently rinsed with PBS to remove unbound cells. The coverslips were subsequently placed onto microscope slides and processed using the method adopted from Allison and Sutherland [109]. Coverslips were covered with 10 mM cetylpyridinium chloride and air-dried before heat fixation. The biofilms were then stained for 15 min with a 2:1 mixture of saturated Congo red solution (Sigma-Aldrich) and 10% Tween 20 (Sigma-Aldrich). The slides were then rinsed, counterstained with 10% Ziehl-carbol fuchsin, rinsed again, and dried at 37 °C. The prepared coverslips were viewed under a light microscope. 

### 3.5. Biofilms Grown on Stainless Steel

To observe the biofilms developed on the stainless steel coupons, the fixation method [7] was followed, with some specifications. Figure S2 shows a schematic representation of the stainless-steel coupon treatment for SEM imaging. Briefly, we used V2R stainless steel coupons (0.8 mm thickness, cut into square shapes of approximately 2 × 2 cm^2^), which were previously rinsed with acetone, isopropanol, and deionized water to remove organic contamination and subsequently sterilized by autoclaving procedure (121 °C for 20 min). Stainless steel samples were incubated at 30 °C for 24, 48, and 72 h with the assayed *L. parabuchneri* DSMZ 5987 strain inoculated in MRS. Similarly, the second set of experiments was performed but this time in the inoculated suspension with stainless steel coupons (500 μL *L. parabuchneri* solution in 50 mL MRS broth), and 600 μL of 0.05% chitosan solution were added into suspension. The same protocol is followed for TMC. After incubation, coupons were rinsed with PBS buffer, fixed in 2.5% glutaraldehyde, dehydrated with a graded series of acetone solutions (50–100%), dried process of the samples with argon, and coated with platinum (SDC 005 sputter coater). SEM measurements were performed using a dual-beam FIB/SEM system (Quanta 3D FEG, FEI Company, Eindhoven, NL, USA). 

### 3.6. IR-ATR Flow System for Biofilm Monitoring

Infrared-attenuated total reflection (IR-ATR) spectroscopy was used to examine the behavior of *L. parabuchneri* biofilms. As shown in the present study, this method provides unprecedented insights into the initial attachment of *L. parabuchneri* at the surface and allows for the study of the temporal behavior of *L. parabuchneri* biofilms near the waveguide surface. In this study, a customized horizontal ATR flow cell assembly composed of a bottom stainless-steel plate for mounting on a trapezoidal ZnSe crystal and a customized removable plate was developed. The top plate was fabricated by a machinery shop at Ulm University from polyether ether ketone (PEEK) and modified to precisely position mounts of polymer optical fibers between the IR-active sensing locations and tubing for the inlet and outlet of the solutions.

A flow cell with an internal volume of 1.75 mL and a surface contact area of the ZnSe crystal of 5.2 cm^2^ was clamped onto the surface of the IRE and sealed using a rubber gasket. The flow cell assembly was placed in the sample chamber of a Tensor II Fourier transform infrared (FT-IR) spectrometer (Bruker Optics, Etlingen, Germany) equipped with a DTGS detector and a six-reflection ATR ZnSe crystal (dimensions of approximately 72 mm × 10 mm × 6 mm at the top side). A peristaltic pump (Watson Marlow Series 400; Cornwall, UK) was used to circulate the bacterial and feed solutions through the system. All solutions flowed across the surface of the IRE at a flow rate of 0.78 mL/min, resulting in a residence time of 150 s within the flow cell. The flow rate was periodically monitored by counting the drops at the sample outlet. The growth medium from the inoculation flask was pumped into the flow cell by a peristaltic pump and then into a waste reservoir, which was via silicone tubing connected to the system via Luer-lock assemblies. In this way, the complete setup for monitoring the biochemical composition of *L. p* biofilms was functionalized. Prior to in situ measurements, the crystal was cleaned in a UV light chamber using intense ultraviolet light. Subsequently, the mounted cell was cleaned with ethanol for half an hour. The ATR flow cell was dismantled and cleaned with a 4% (*v*/*v*) Korsolex solution and sterilized water for 20 min at 2.2 a flow rate. Afterwards, the MRS medium was allowed to flow for 120 min at 0.78 a flow rate to ensure the formation of a conditioning film. The MRS conditioning film was recorded as the background spectrum for subsequent *L. parabuchneri* biofilms to minimize water interference. The *L. parabuchneri* bacterial solution (1 × 10^9^ CFU/mL) was introduced for 4 h at a flow rate 0.78 mL/min. The inlet was switched to the MRS medium overnight. The chitosan stock solution was inserted into the flow system after 24 h of *L. parabuchneri* biofilm formation. The inlet was switched from time to time to the MRS media at 0.7 a flow rate and IR-flow measurements were recorded for 168 h. The same protocol was followed for TMC experiments. Prior to each biofilm-monitoring experiment, the entire system was disinfected with 70% ethanol for several hours and then rinsed with sterile water to ensure complete removal of ethanol. All glassware and syringes were sterilized in an autoclave at 121 °C (15 min, 3 bar, Systec VE-150). 

### 3.7. Integration of IR Spectroscopy and Fluorescence Sensing

Quantitative determination of oxygen is an important parameter in food packaging, bioprocessing control, and industrial production monitoring. Optical oxygen sensors applicable in liquid- and gas-phase measurements, which are not influenced by the flow rate of the sample and have excellent long-term stability, were integrated into the IR-ATR flow cell. Optical analysis of oxygen is typically based on the effect of dynamic luminescence quenching by molecular oxygen [69,71,110]. The fundamental principles are illustrated in Figure S4. Upon light absorption, the luminophore is excited and emits light in the absence of oxygen (Figure S4, left). In the presence of molecular oxygen, which acts as a quencher, the collision between the luminophore in its excited state and oxygen leads to nonradiative deactivation (Figure S4, right).

The frequency of collisions correlates with the degree of fluorescence quenching and therefore with the concentration, pressure, and temperature of the oxygen-containing medium. Almost all long-lived fluorophores dissolved in organic solvents can be used as oxygen sensors. In this study, oxygen sensor spots composed of platinum (II)- 5,10,15,20- tetrakis-(2,3,4,5,6-pentafluorophenyl) porphyrin (PtTFPP) were used owing to their excellent response to oxygen [69,71,110]. These circular sensor spots were then immobilized within the IR-inactive sensing areas at the ZnSe crystal surface without the use of glue by taking advantage of the rubber gasket to hold them in place. Previous studies have shown the localization of IR-active sensing regions [67], helping us to determine the IR-inactive areas along the crystal surface. 

Commercial contactless oxygen sensors, known as retractable needle-type oxygen microsensors, and flow-through cells with integrated oxygen sensors were obtained from PyroScience GmbH (Aachen, Germany). FireStingO2 enables 4-channel oxygen measurement to transmit data from oxygen sensors via a USB interface to a personal computer. This oxygen meter excites the dye with red light (wavelength of 610–630 nm) and measures the oxygen-dependent luminescence in the NIR (760–790 nm) (Figure 2). After each measurement, the sensor spots were cleaned by rinsing them with 70% ethanol and demineralized water. The spots were stored at room temperature and protected from the ambient light. The oxygen concentration in the gas and liquid phases (mg/mL) was measured every 5 min during real-time IR-ATR monitoring of the biofilms.

The metabolic activity of pH changes was monitored to evaluate the efficiency of chitosan and TMC inhibition in *L. parabuchneri* biofilms. The pH was measured prior to inoculation of MRS media and *L. parab.* solution into the flow system after the flow process is completed. The remaining biofilm suspension mixed with MRS broth into the flow cell was used for pH investigation. pH measurements were performed using a calibrated Metrohm pH meter (Deutsche Metrohmn GmbH &Co. KG, Filderstadt, Germany).

## 4. Conclusions

The present study indicates strong antimicrobial effects of chitosan and its methylated derivative on *Lentilactobacillus parabuchneri* DSMZ 5987 based biofilms, revealed via real-time IR-ATR studies, providing molecular insight into biofilm inhibition. The obtained molecular information may be deconvoluted using appropriate multivariate data evaluation strategies, along with spectral modeling procedures via cumulative impulse fit approaches. Combined, the results provide a promising tool for in-depth insight into the molecular mechanisms involved in *L. parabuchneri* biofilm inhibition and growth processes associated with spectral features, and changes in vital parameters such as oxygen concentration with cellular biochemical, and structural changes induced by the antimicrobial bioagents chitosan and TMC. IR studies suggested that both the biopolymer chitosan and its derivative provided distinct antimicrobial effects and reduced bacterial proliferation, with a remarkable decrease in EPS matrices. This confirms that efficient disruption of the cell wall is detrimental to the bacterial communities. Minimum inhibitory concentration (MIC) plays an important role in antimicrobial activity against biofilm formation. The tested MIC values showed promising bioactivity despite the comparative results obtained between chitosan and TMC. The antibacterial activities of chitosan and its water-soluble derivative, trimethyl chitosan (TMC), were evaluated using optical microscopy and SEM. The obtained results confirmed that mature biofilms progressively turned into detached microcolonies, which were delaminated from the stainless steel and glass coverslip surfaces owing to the disintegration of the biofilm matrix. Finally, a quantitative microtiter plate assay was used to investigate the inhibitory effect of chitosan and TMC on biofilm formation after 24, 48, and 72 h and their ability to inhibit bacterial adhesion and biofilm formation by up to 50%. The morphological SEM and light microscopy investigations of stainless steel and glass coverslip coupons pretreated with chitosan and its methylated derivative (TMC) showed beyond a doubt that the inhibition efficiency by structural changes in *L. parabuchneri* biofilms are due to due to destructive effects of chitosan compounds, so the biofilms seem to transform into isolated microcolonies which peel off from the surface of coverslips. This proves that chitosan and TMC can be considered as an alternative for efficient bioderived antimicrobials with a significant interest to the food production and post-ripening processing in dairy industries. Chitosan and its derivative TMC are safe natural antimicrobials as an alternative to be used in the food packaging industry to prevent biofilm formation of cheese contaminant *L. parabuchneri.*

## Figures and Tables

**Table 1:** Restriction of EPS production peak parameters and expected peak attributions in the spectral region from 1150–950 cm^−1^ of *Lentilactobacillus parabuchneri* DSMZ 5987 + MRS biofilm inhibition were used to evaluate the cumulative impulse fit of polysaccharides by the influence of chitosan and its TMC derivative.

Peak Attribution	x_0_ [cm^−1^]	w Range [cm^−1^]
P-OH/C-C ($\bar{x}=1108.1{\;\mathrm{cm}}^{-1})\;$ [84,86]	1108.3	5–45
ν P = O ($\bar{x}=1071.1{\;\mathrm{cm}}^{-1}$) [50,84,85]	1079.8	5–30
ν O–H/δ C–O ($\bar{x}=1040.6{\;\mathrm{cm}}^{-1}$) [86]	1038.9	5–35
Ν phosphoryl group ($\bar{x}=991.1{\;\mathrm{cm}}^{-1}$) [50]	993.0	5–50

## Data Availability

Not applicable.

## Supplementary Materials

The following supporting information can be downloaded at: https://www.mdpi.com/article/10.3390/molecules27248647/s1, Table S1: Oxygen level values measured by optical fiber oxygen flow-microsensor during IR-ATR biofilm formation for 48 h. The values are presented as the mean of three replicates ± standard deviation (SD), Figure S1: PCA score values for the models of variance build upon IR datasets of *L. parabuchneri* biofilm inhibition from chitosan and its quaternized derivative TMC, Figure S2: Schematic representation of stainless-steel treatment prior performance of SEM measurements, Figure S3: Disintegration of the biofilm matrix into individual cells as a result of inhibition efficiency by the addition of (**a**) 0.05% Chitosan stock solution and (**b**) TMC derivative. Red circles correlate with separated bacterial cells and Figure S4: Fundamentals of fluorescence quenching in the presence of oxygen as a quencher causing collision [69,71,110].

## Sample Availability

Samples of the compounds presented in this study are available upon reasonable request from the first and corresponding author.
