# Supplementary material for: Antimicrobial Efficiency of Chitosan and Its Methylated Derivative against Lentilactobacillus parabuchneri Biofilms
Source: Molecules. 2022 Dec 7;27(24):8647. doi: 10.3390/molecules27248647 (PMC9786053; doi:10.3390/molecules27248647)
Supplement: Supplementary file 1 [file molecules-27-08647-s001.zip › molecules-2051025-supplementary.pdf]

Supplementary Materials

Antimicrobial efficiency of chitosan and its methylated derivative against *Lentilactobacillus parabuchneri* biofilms

Diellza Bajrami[1]**,** Stephan Fischer[2], Holger Barth[2], Syed Imdadul Hossain[3,4], Nicola Cioffi[3,4]**, Boris Mizaikoff**[1,5*]

[1] Institute of Analytical and Bioanalytical Chemistry, Ulm University, Albert Einstein-Allee 11, 89081 Ulm, Germany
[2] Institute of Pharmacology and Toxicology, Ulm University Medical Center, Albert Einstein-Allee 11, 89081 Ulm, Germany
[3] Chemistry Department, University of Bari ''Aldo Moro'', V. Orabona, 4, 70126, Bari, Italy
[4] CSGI (Center for Colloid and Surface Science) c/o Chemistry Department, via E. Orabona, 4, 70126 Bari, Italy
[5] Hahn-Schickard, Institute for Microanalysis Systems, Sedanstrasse 14, 89077 Ulm, Germany
* Correspondence: boris.mizaikoff@uni-ulm.de; Tel.: +49-731-50-22750 (B.M)

Table S1. Oxygen level values measured by optical fiber oxygen flow-microsensor during IR-ATR biofilm formation for 48 h. The values are presented as the mean of three replicates ± standard deviation (SD).

Time/min	O_2 levels during *L. p.* biofilm inhibition by chitosan (mg/mL)	O_2 levels during *L. p.* biofilm inhibition by TMC (mg/mL)
**2**	4.02 ± 0.3	4.46 ± 0.5
**4**	3.78 ± 0.6	3.74 ± 0.7
**6**	3.68 ± 0.5	3.52 ± 0.3
**8**	3.61 ± 0.3	3.4 ± 0.6
**10**	3.54 ± 0.3	3.35 ± 0.8
**12**	3.48 ± 0.6	3.1 ± 0.5
**14**	3.33 ± 0.5	2.91 ± 0.6
**16**	3.27 ± 0.7	2.61 ± 0.3
**18**	3.11 ± 0.9	2.43 ± 0.7
**20**	3.14 ± 0.3	2.31 ± 0.7
**22**	3.04 ± 0.5	2.03 ± 0.6
**24**	2.9 ± 1.0	1.98 ± 0.5
**26**	2.7 ± 0.6	1.9 ± 0.7
**28**	2.68 ± 0.8	1.79 ± 0.5
**30**	2.3 ± 1.0	1.64 ± 0.3
**32**	2.1 ± 0.5	1.52 ± 0.8
**34**	1.92 ± 0.5	1.42 ± 0.5
**36**	1.78 ± 0.2	1.35 ± 0.6
**38**	1.54 ± 0.3	1.2 ± 1.5
**40**	1.42 ± 0.8	1.16 ± 0.6
**42**	1.3 ± 0.7	1.08 ± 0.2
**44**	1.27± 0.2	1 ± 0.7
**46**	1.13 ± 0.4	0.98 ± 0.4
**48**	1 ± 0.6	0.95 ± 0.5

1. Chemometric modeling

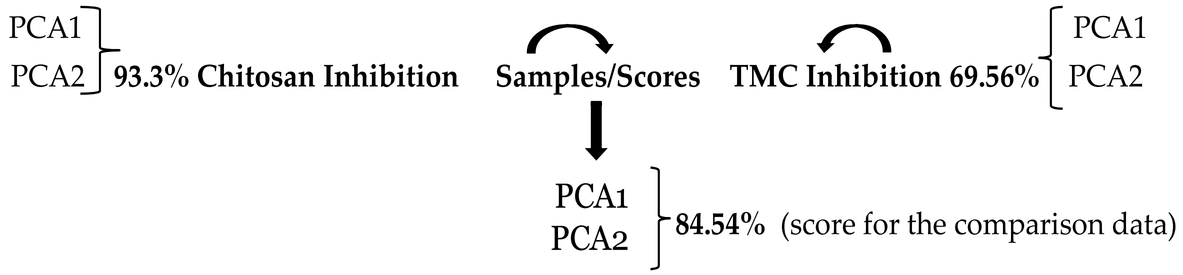

Figure S1. PCA score values for the models of variance build upon IR datasets of *L. parabuchneri* biofilm inhibition from chitosan and its quaternized derivative TMC.

2. SEM studies

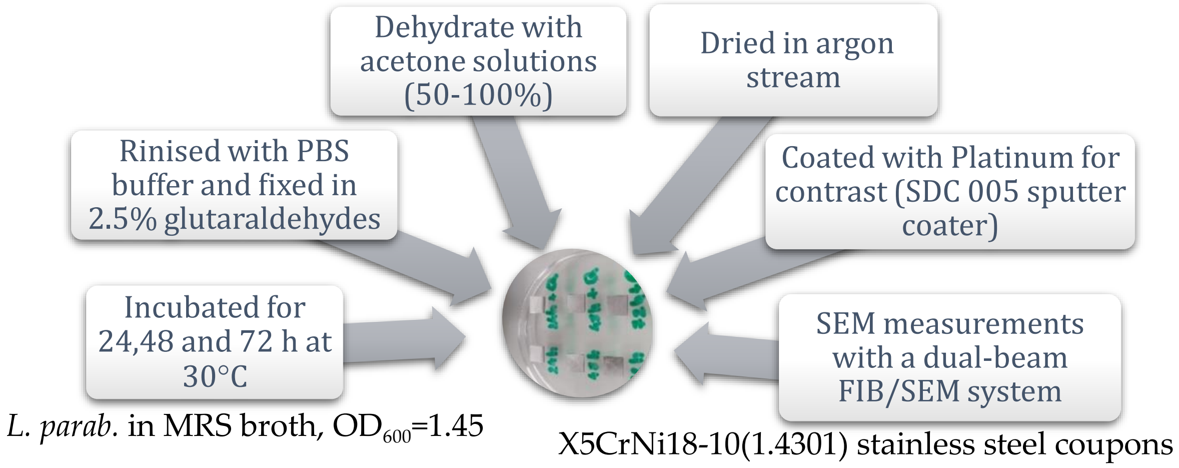

Figure S2. Schematic representation of stainless-steel treatment prior performance of SEM measurements.

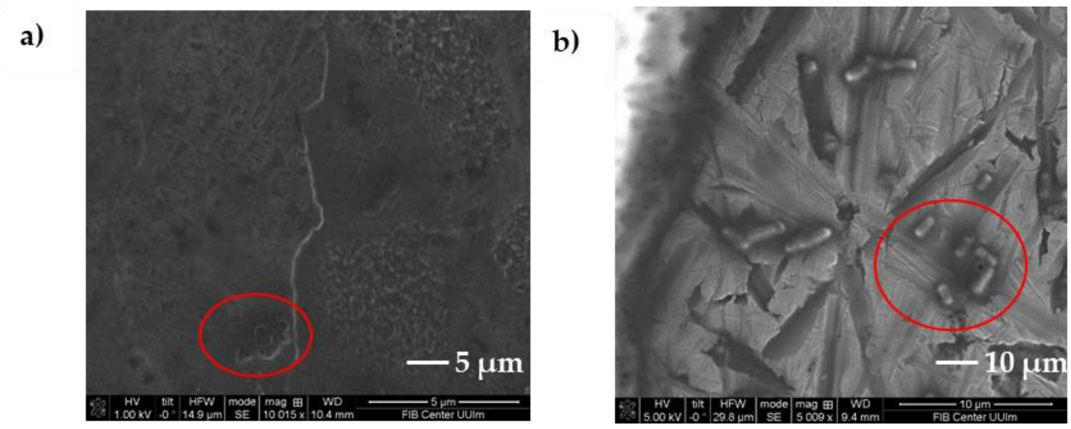

Figure S3. Disintegration of the biofilm matrix into individual cells as a result of inhibition efficiency by the addition of **a)** 0.05 % Chitosan stock solution and **b)** TMC derivative. Red circles correlate to separated bacterial cells.

3. **Fluorescence sensing**

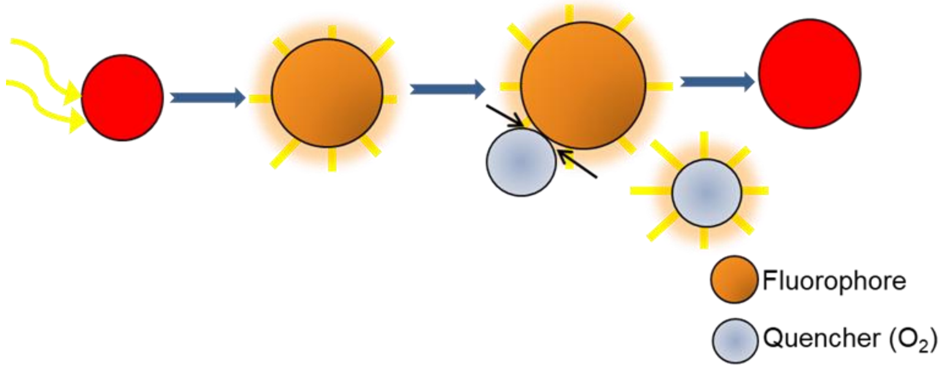

Figure S4. Fundamentals of fluorescence quenching in the presence of the oxygen as a quencher causing collision.

The degree of fluorescence quenching depends on the concentration, pressure, and temperature of the oxygen-containing medium. Almost all long-lived fluorophores dissolved in organic solvents can be used as oxygen sensors. Oxygen sensor spots used in this study were composed of platinum (II)- 5,10,15,20- tetrakis-(2,3,4,5,6-pentafluorophenyl) porphyrin (PtTFPP) with an excellent oxygen response [69,71,110].

References

69. Ganesh, A.B.; Radhakrishnan, T.K. Fiber-Optic Sensors for the Estimation of Oxygen Gradients within Biofilms on Metals. *Opt. Lasers Eng.* **2008**, *46*, 321–327. https://doi.org/10.1016/j.optlaseng.2007.11.003.

71. Scheicher, S.R.; Kainz, B.; Köstler, S.; Suppan, M.; Bizzarri, A.; Pum, D.; Sleytr, U.B.; Ribitsch, V. Optical Oxygen Sensors Based on Pt(II) Porphyrin Dye Immobilized on S-Layer Protein Matrices. *Biosens. Bioelectron.* **2009**, *25*, 797–802. https://doi.org/10.1016/j.bios.2009.08.030.

110. McDonagh, C.; Kolle, C.; McEvoy, A.K.; Dowling, D.L.; Cafolla, A.A.; Cullen, S.J.; MacCraith, B.D. Phase Fluorometric Dissolved Oxygen Sensor. *Sens. Actuators B Chem.* **2001**, *74*, 124–130. https://doi.org/10.1016/S0925-4005(00)00721-8
